# Deep CNN models for predicting COVID-19 in CT and x-ray images

**DOI:** 10.1117/1.JMI.8.S1.014502

**Published:** 2021-04-21

**Authors:** Ahmad Chaddad, Lama Hassan, Christian Desrosiers

**Affiliations:** a Guilin University of Electronic Technology, School of Artificial Intelligence, Guilin, China; b University of Quebec, Ecole de Technologie Supérieure, Montreal, Canada

**Keywords:** convolutional neural network, Coronavirus disease 2019, transfer learning, radiomics

## Abstract

**Purpose:** Coronavirus disease 2019 (COVID-19) is a new infection that has spread worldwide and with no automatic model to reliably detect its presence from images. We aim to investigate the potential of deep transfer learning to predict COVID-19 infection using chest computed tomography (CT) and x-ray images.

**Approach:** Regions of interest (ROI) corresponding to ground-glass opacities (GGO), consolidations, and pleural effusions were labeled in 100 axial lung CT images from 60 COVID-19-infected subjects. These segmented regions were then employed as an additional input to six deep convolutional neural network (CNN) architectures (AlexNet, DenseNet, GoogleNet, NASNet-Mobile, ResNet18, and DarkNet), pretrained on natural images, to differentiate between COVID-19 and normal CT images. We also explored the model’s ability to classify x-ray images as COVID-19, non-COVID-19 pneumonia, or normal. Performance on test images was measured with global accuracy and area under the receiver operating characteristic curve (AUC).

**Results:** When using raw CT images as input to the tested models, the highest accuracy of 82% and AUC of 88.16% is achieved. Incorporating the three ROIs as an additional model inputs further boosts performance to an accuracy of 82.30% and an AUC of 90.10% (DarkNet). For x-ray images, we obtained an outstanding AUC of 97% for classifying COVID-19 versus normal versus other. Combing chest CT and x-ray images, DarkNet architecture achieves the highest accuracy of 99.09% and AUC of 99.89% in classifying COVID-19 from non-COVID-19. Our results confirm the ability of deep CNNs with transfer learning to predict COVID-19 in both chest CT and x-ray images.

**Conclusions:** The proposed method could help radiologists increase the accuracy of their diagnosis and increase efficiency in COVID-19 management.

## 1 Introduction

In December 2019, a new coronavirus disease, called COVID-19 by the World Health Organization,^1^ was discovered in Wuhan, Hubei, China. This viral infection, for which there is no effective treatment to date, spread quickly across and outside China, causing severe acute respiratory syndrome (SARS) in the infected population.^2^ In March 2020, the crisis reached the pandemic stage as the worldwide outbreak accelerated.^3^ Many techniques have been used to estimate and identify the presence of COVID-19, including measuring body temperature, reverse-transcription-polymerase chain reaction (RT-PCR), chest computed tomography (CT)-scan, and chest x-ray.^4^^–^^7^ Unfortunately, body temperature is not an accurate biomarker and molecular analysis techniques (e.g., blood-routine and infection-biomarkers) are not only costly but also need high processing times. Moreover, they can potentially have serious side effects such as secondary infection. The RT-PCR test, which is widely used for confirming COVID-19 infection, can also lead to false negatives. Hence, two studies in Refs. 8 and 9 found that 3% to 30% of COVID-19 patients who initially had a negative RT-PCR test showed a positive chest CT a few days later, this infection was then confirmed by a second RT-PCR. Given the low sensitivity of the RT-PCR test,^10^ automated and reliable methods to screen COVID-19 patients are required. Medical imaging techniques, such as chest CT and chest x-ray, offer a noninvasive alternative to identify COVID-19.^11^^–^^14^ However, clinicians are not always able to identify small changes within scans/images caused by the presence of COVID-19. Therefore, there is a pressing need for intelligent tools to predict COVID-19 infection from medical images.

Imaging features derived from CT can describe characteristics of infected tissues^15^ and have been used for detecting the presence of COVID-19. Several recent works have investigated the usefulness of CT imaging features to distinguish COVID-19 from other viral infections.^16^^–^^20^ It was shown, unfortunately, that COVID-19 produces CT features similar to those caused by pneumonia.^17^ Moreover, the study in Ref. 21 reported that COVID-19 can mimic diverse disease processes, including other infections, which can lead to a misdiagnosis between COVID-19 and other viral pneumonia. It is argued that the automatic classification between COVID-19 and other types of pneumonia could avoid unnecessary efforts and decrease the spread of COVID-19 infection. Also, Wong et al.^22^ studied the appearance of COVID-19 in chest x-ray, and its correlation with key findings in CT scans and RT-PCR tests. To date, only a few studies have considered imaging features obtained from deep learning models for predicting, detecting, and screening COVID-19.

Machine learning techniques have recently led to a paradigm shift in analyzing complex medical data. In particular, deep learning algorithms such as the convolutional neural network (CNN) have shown an outstanding ability to automatically process large amounts of medical images, and to identify complex associations in high-dimensional data for disease diagnosis and treatment planning.^23^ Radiomics analysis, which extracts high-throughput features from medical images and uses them for multiple clinical prediction tasks, has had a high impact in medical image analysis and computer-aided diagnosis.^24^ For instance, radiomics models based on CT and x-ray have been proposed for predicting pneumonia associated with SARS-CoV-2 (COVID19) infection^25^^–^^27^ and for assisting clinical decision making.^28^ Recently, deep learning algorithms were successfully applied on CT and x-ray images for the automated detection of COVID-19^14^^,^^27^^,^^29^^–^^31^ and for classifying bacterial from viral pneumonia in pediatric chest radiographs.^32^ Moreover, many studies have shown the usefulness of CT features related to COVID-19 [e.g., ground-glass opacities (GGO), mixed GGO and consolidation, and subpleural lesions].^33^^,^^34^ Despite their achievements, more investigation is needed to analyze separately the impact of imaging features derived from specific regions of interest (ROI) in CT, namely, GGO, consolidation and pleural effusion (PE), in predicting COVID-19.

While recent work^35^ has shown the advantage of deep CNNs for predicting clinical outcomes compared to traditional radiomic pipelines, the direct application of such strategy is also prone to overfitting when few labeled examples are available, leading to poor generalization on new data. To overcome the problem of limited training data, the work in Ref. 36 proposed using entropy-related features extracted at different layers of a CNN to train a separate classifier model for the final prediction. The approach of this previous work is based on the principle of transfer learning, where convolutional features learned for a related image analysis task can be reused to improve the learning of a new task. This technique is well-suited for detecting anomalies such as lesions in medical images since those anomalies are typically characterized by local changes in texture and not high-level structures in the image. Therefore, low-level features in the network, capturing general characteristics of texture, can be transferred across different image analysis tasks. However, an important limitation of this work is that it summarized CNN features in a very limited number of texture descriptors and it only considered a single network architecture. This study presents a deep transfer learning (DTL) approach to predict COVID-19 infections from abnormal chest CT and x-ray images. Specifically, we propose to exploit features learned from six different deep CNN architectures and boost DTL models using the ROIs in addition to the training images for predicting COVID-19. We hypothesize that pre-training these networks on a large dataset of images with confirmed COVID-19 ROIs can help to learn informative features that capture local texture anomalies related to COVID-19 infections. Moreover, we demonstrate that analyzing the distribution of these features within ROIs corresponding to distinct findings can yield a high accuracy for discriminating between COVID-19 and other types of pneumonia. The main contributions of our work are the following.

1. We propose a DTL approach that learns image features capturing tissue heterogeneity, which can effectively predict COVID-19 infection with limited training data.
2. To the best of our knowledge, this is the first work to analyze deep features by integrating separate CT lung ROIs images (i.e., GGO, consolidation and PE) in DTL models.
3. We present a comprehensive analysis of DTL for COVID-19 prediction, involving several datasets of different modalities and six deep CNN architectures. Our results demonstrate the potential of the proposed approach for differentiating between COVID-19 and other viral pneumonia.

The rest of this paper is structured as follows. Section 2 describes the data used in this study, as well as the proposed pipeline based on DTL. We then present the experimental results in Section 3 and discuss our main findings in Sec. 4. Finally, Sec. 5 concludes with a summary of our work’s main contributions and results.

## 2 Materials and Methods

**Fig. 1:**
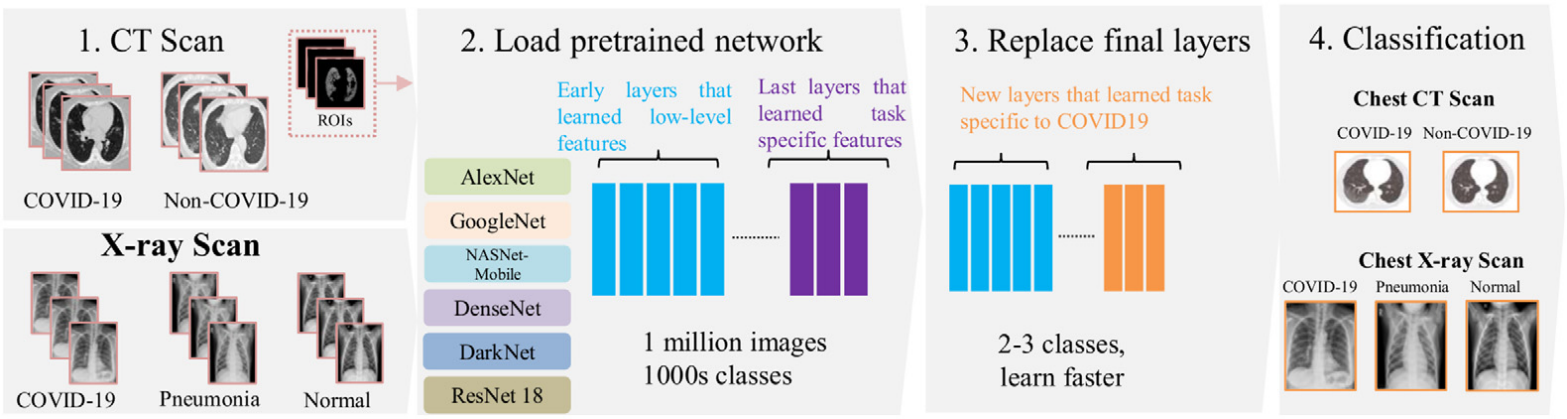
A proposed pipeline for predicting the COVID-19 using the CT and x-ray images with deep transfer learning models. (1) Image acquisition of axial CT scans (or x-ray images) with semi-automatic labeling of lung lesions ROIs (GGO, consolidation, and PE); (3) and (4) six pretrained CNNs models were considered and the last layers were adapted (replaced) to predict COVID-19.

Figure 1 shows the pipeline to detect the presence of COVID-19 in CT and x-ray images. First, images are acquired by a CT or x-ray scanner. One hundred axial CT images are then segmented in a semi-automatic fashion using the MedSeg tool^37^ to label ROIs corresponding to three types of findings: GGO, consolidation, and PE to then add to the main CT images used in the DTL training. X-ray images are used without segmentation. For transfer learning, six well-known CNN models are considered: AlexNet, DenseNet, GoogleNet, NASNet-Mobile, ResNet18, and DarkNet. These networks were pretrained on a large dataset for image classification and are adapted to the target tasks by retraining only the final layers of the architecture. Models are evaluated on three prediction tasks: (1) classifying COVID-19 versus non-COVID-19 CT scans, (2) classifying COVID-19 versus normal x-ray images, and (3) classifying COVID-19 versus other viral pneumonia in x-ray images.

### 2.1 Patients and Data Acquisition

Our study uses a total of 846 (COVID-19 = 349, non-COVID-19 = 397, and COVID-19 ROIs = 100) axial CT slice images. The COVID-19 datasets have 349 CT images containing clinical findings of COVID-19 from 216 patients. These 349 COVID-19 CT images were selected by a senior radiologist in Tongji Hospital, Wuhan, China, during the outbreak of this disease between January and April 2020 (https://github.com/UCSD-AI4H/COVID-CT). More details about these 349 COVID-19 images are described in Ref. 38.

Moreover, we collected a set of 397 non-COVID-19 CT slice images from 397 patients (36 from Lung Nodule Analysis,^39^ 195 from MedPix,^40^ 136 from PubMed Central,^41^ and 30 from Radiopaedia^42^) as detailed in Ref. 38. To tune our DTL models, we used another set of 100 labeled slice images (e.g., GGO, Consolidation, and PE) from 60 COVID-19 patients, obtained from the COVID-19 radiology-Data Collection and Preparation for Artificial Intelligence, the Italian society of medical and interventional radiology (SIRM).^43^ These labeled images have been previously de-identified by radiologists and, therefore, no institutional review board or Health Insurance Portability and Accountability Act approval was required for our study. Details on the acquisition protocol can be found in Ref. 44. As noted in labeling the ROIs, images were in the format of JPG, resized to 512×512  pixels, converted to grayscale and then compiled into a single NIFTI-file. The segmentation was performed by radiologists using the MedSeg tool^37^ to delineate ROIs corresponding to GGO, consolidation and PE findings. In some cases, a label of whole abnormal tissue was used for findings that did not fit in one of the three ROI categories.

**Fig. 2:**
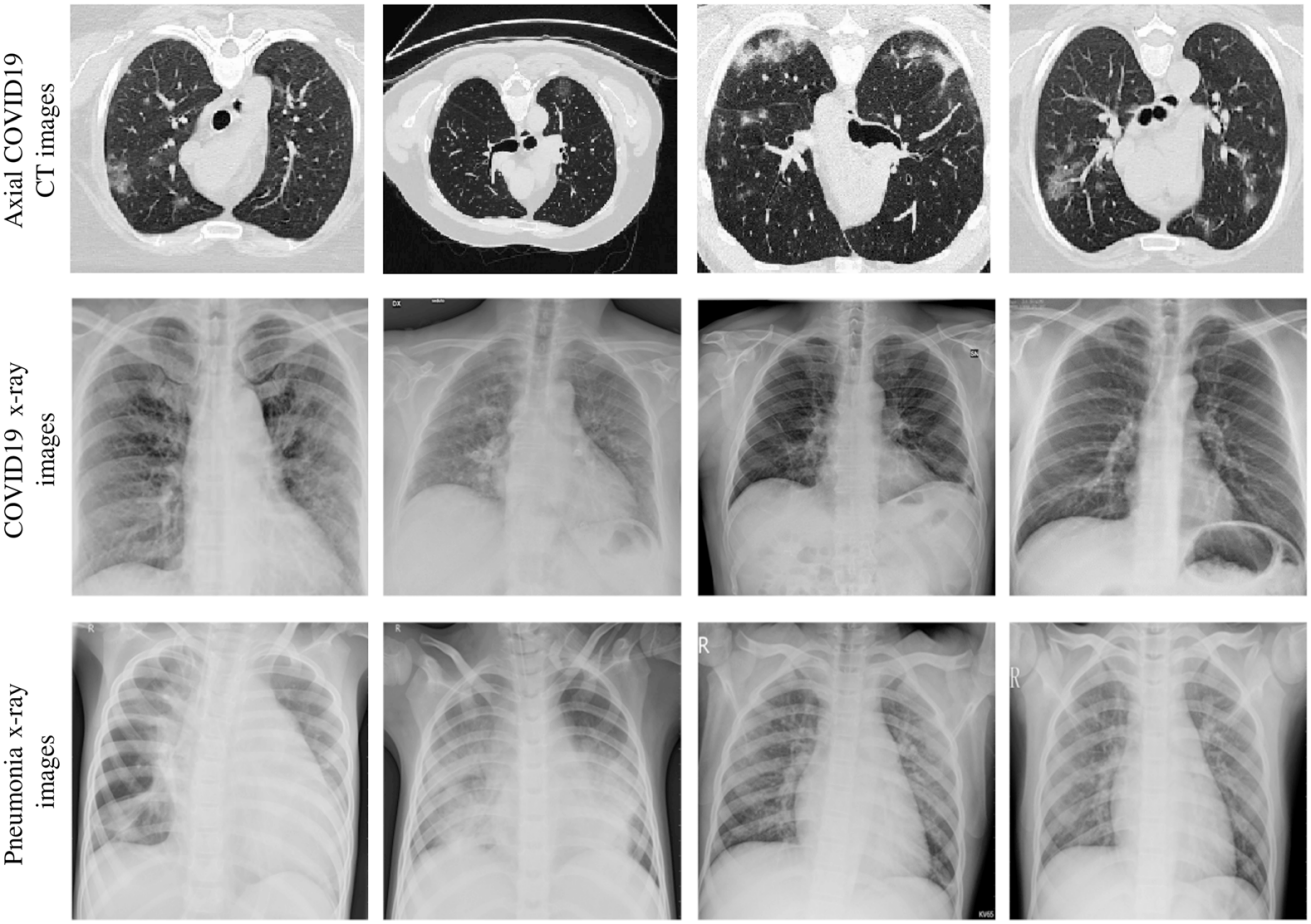
Examples of COVID-19 in CT and x-ray images. First row: axial COVID-19 CT images with lesions in different positions and sizes. Second row: COVID-19 x-ray images. Third row: pneumonia x-ray images.

Moreover, our study also leverages 657 chest x-ray slice images collected from multiple sources: 219 x-ray images of COVID19-infected patients from the COVID chest x-ray Dataset (http://github.com/ieee8023/covid-chestxray-dataset), the SIRM, Radiopaedia, and the Radiological Society of North America;^45^ 219 normal (subjects) and 219 patients with pneumonia (i.e., viral and bacterial) x-rays from a publicly available Kaggle dataset.^32^^,^^46^^,^^47^ These chest x-ray slice images were in the format of JPG obtained from multisite with various scanner models, pixel spacing, and contrast. Thus, we considered these differences by sampling these entire images to a common resolution (i.e., $1\;{\mathrm{mm}}^{2}$) with a size of 512×512. All the obtained CT and x-ray images were also normalized to the [0; 255] range. Figure 2 shows examples of COVID-19 chest CT images, and x-ray images of COVID-19 and non-related pneumonia.

### 2.2 Deep Convolutional Neural Networks

Deep CNNs have demonstrated an impressive performance for various image classification tasks, in particular when large sets of images are available.^27^^,^^28^ Various CNN architectures have been proposed for different applications in computer vision,^48^ big data, and biomedical imaging.^49^ At a high level, CNN architectures comprise a repeated stack of convolution and pooling layers, followed by one or more fully connected layers.^50^ Convolution layers apply a filtering function to extract spatial features from an input image. These features encode different levels of abstraction, with initial layers capturing local image patterns and texture, and deeper layers extracting high-level features representing the global structure. To add non-linearity, a non-saturating activation function such as the rectified linear unit^30^ is typically employed. Such function helps alleviate the vanishing gradient problem when training deep networks. Pooling layers (e.g., maximum or average) are typically added after each convolution layer block to reduce the spatial dimension of feature maps and make the network invariant to small image translations. CNNs for classification also have fully connected layers at the end of the network, followed by an output layer (e.g., softmax), which converts logits into class probabilities. During training, convolutional filters and fully connected layer weights are updated using the backpropagation algorithm.

### 2.3 Proposed Transfer Learning Approach

Transfer learning is a powerful strategy that enables to quickly and effectively train of deep neural networks with a limited amount of labeled data. The basic idea of this strategy is to use a pretrained network on a large available dataset, and then use the features of this network as a representation for learning a new task without re-training from scratch. Transferred features can be used directly as input to the new model or adapted to the new task via fine-tuning.

Following this strategy, our method uses six well-known CNN architectures, i.e., AlexNet, GoogleNet, NASNet-Mobile, DenseNet, DarkNet, and ResNet18, pretrained for image classification on the ImageNet dataset. This dataset contains over 14 million natural images belonging to about 20 thousand categories.^51^ Although the CT and x-ray images in our study are very different from those in this dataset, we argue that relevant information for detecting COVID-19 lies in local changes in texture and that this information can be captured effectively with a general set of low-level features. For adapting these pretrained networks to the task of differentiating between COVID-19 and pneumonia or normal lung images, we replace all layers following the last convolution block (i.e., fully connected and softmax) by new layers of the correct size (e.g., 2 CT image classes and/or 3 x-ray image classes), and fine-tune the modified networks using training examples of the new tasks.

For training, we randomly initialized the weights of fully connected layers and employed stochastic gradient descent with momentum to update all network parameters. We set the batch size to 10, the learning rate to $1\times 10^{-4}$, and the number of epochs to 10. The dataset was split into three independent subsets containing different subjects, with ∼70% (429 CT-patients; 460 x-ray patients), ∼10% (61 CT-patients; 66 x-ray patients), and ∼20% (123 CT-patients; 131 x-ray patients), of examples for training, validation, and testing, respectively. To prevent overfitting,^52^ we augmented the training dataset using the following image transformations: random flipping, rotation, translation, and scaling.

### 2.4 Evaluation Metrics

The performance of tested models was evaluated on test images, using the area under the curve (AUC) of the receiver operator characteristic (ROC) curve, accuracy, and confusion matrix. We measured performance separately for the prediction tasks using CT and x-ray images. The statistical significance of the difference in performance was assessed using the Wilcoxon test.^53^ For multiple comparisons, we considered the Holm–Bonferroni method in correcting the obtained p-values.^54^ All processing/analysis steps were performed using MATLAB’s deep learning, statistics, and machine learning toolbox.

## 3 Results

As mentioned before, our experiments use a dataset of 746 CT images (COVID-19 = 349 and non-COVID-19 = 397) from 216 patients and 657 chest x-ray images (219 COVID-19, 219 normal, and 219 pneumonia). To assess the ROIs (i.e., GGO, consolidation, and PE), we combined 100 CT images derived from 60 patients with COVID-19 that have a total of 95 GGO, 80 consolidations and 25 PE finding ROIs.

In Table 1, we observe a test accuracy ranging from 70% to 79% (i.e., AlexNet, GoogleNet and ResNet18) to 80% to 82.80% (i.e., DarkNet, DenseNet, and NASNet-Mobile). The baseline and impact of ROIs in predicting the COVID-19 of each model are also given.

**Table 1:** Accuracy (%) of tested models for classifying COVID-19 versus non-COVID-19 CT images with different finding labels.

CNNs	Testing				
	Baseline	+GGO	+Consolidation	+PE	+Combined
AlexNet	70.00	73.40*	75.90**	73.40*	78.80**
GoogleNet	72.40	75.90*	72.40	72.40	74.40*
DenseNet	**80.80**	79.30	79.30	**80.80**	77.80
NASNet-Mobile	80.30	**82.30**	78.80	80.80	82.30*
DarkNet	82.30	80.80	*82.80*	80.30	82.30
ResNet18	79.00	78.30	79.80	77.80	**80.80**

* significant results with p<0.05

** corrected p-value following Holm–Bonferroni

Note: Bold values represent the maximum value for each of CNN models.

**Fig. 3:**
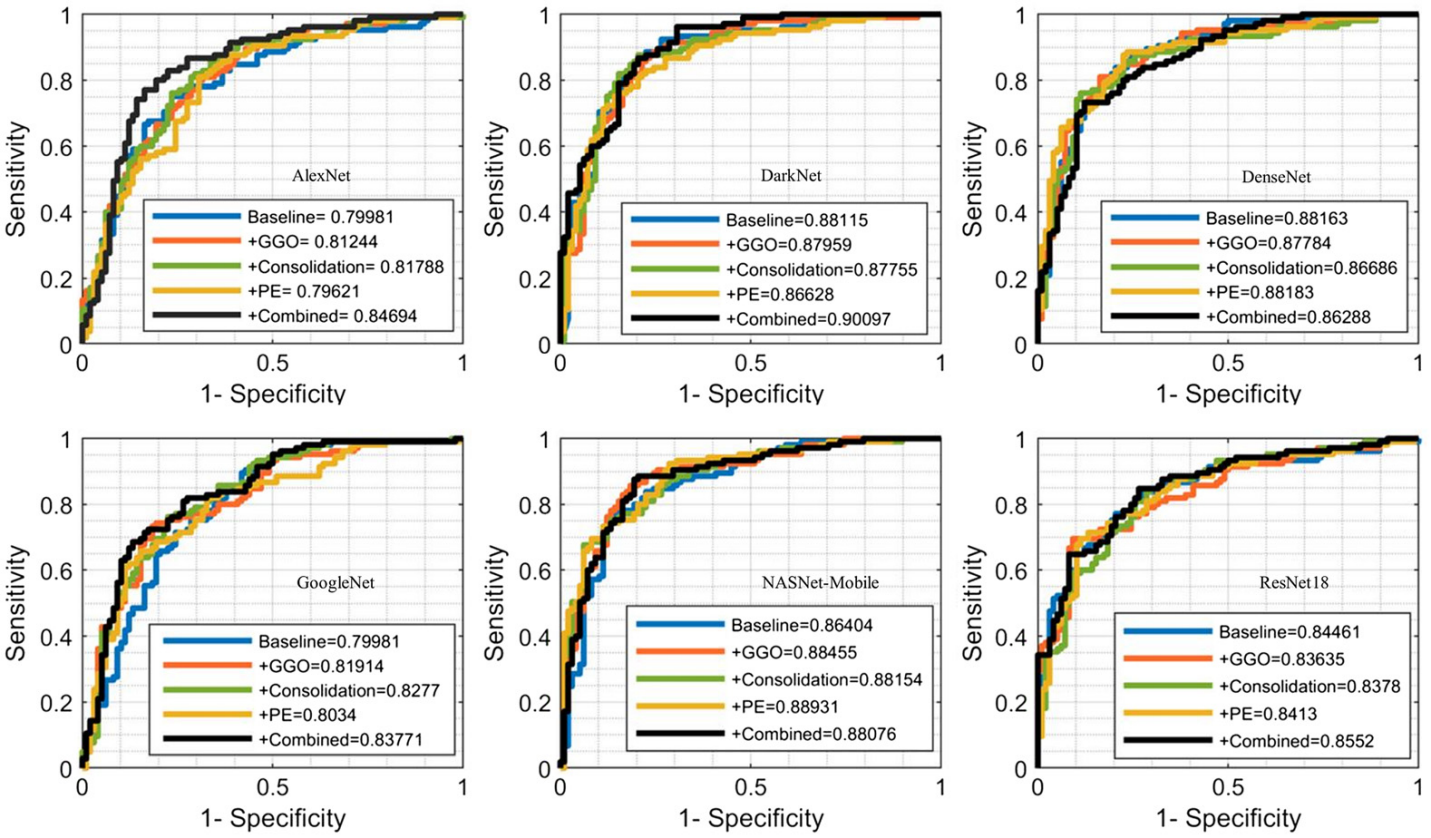
Receiver operating characteristic (ROC)-AUC curve for predicting the COVID-19 CT image using deep transfer learning models.

We find that incorporating GGO ROIs to images (+GGO) improved the accuracy by ∼2% in AlexNet, GoogleNet, and NASNet-Mobile models. On the other hand, combining training images with consolidation (+Consolidation) or PE (+PE) ROIs increased the accuracy only for the AlexNet model. Considered all ROIs together, we found that the accuracy increased using the AlexNet (∼8%), GoogleNet (∼2%), and NASNet-Mobile (∼2%). Next, we computed the AUC-ROC of all six models in predicting the COVID-19 using baseline images, +GGO, +consolidation, +PE, and +Combined ROI labels. The highest AUC value of 90.09% was obtained from DarkNet model using the combined ROIs (Fig. 3). Except for the DenseNet model, we found that AUC increases when we combine baseline with ROIs. When using DarkNet, the highest AUC of 88.45%, 88.15%, and 88.89% is achieved with the +GGO, +consolidation, and +PE combinations, respectively.

**Fig. 4:**
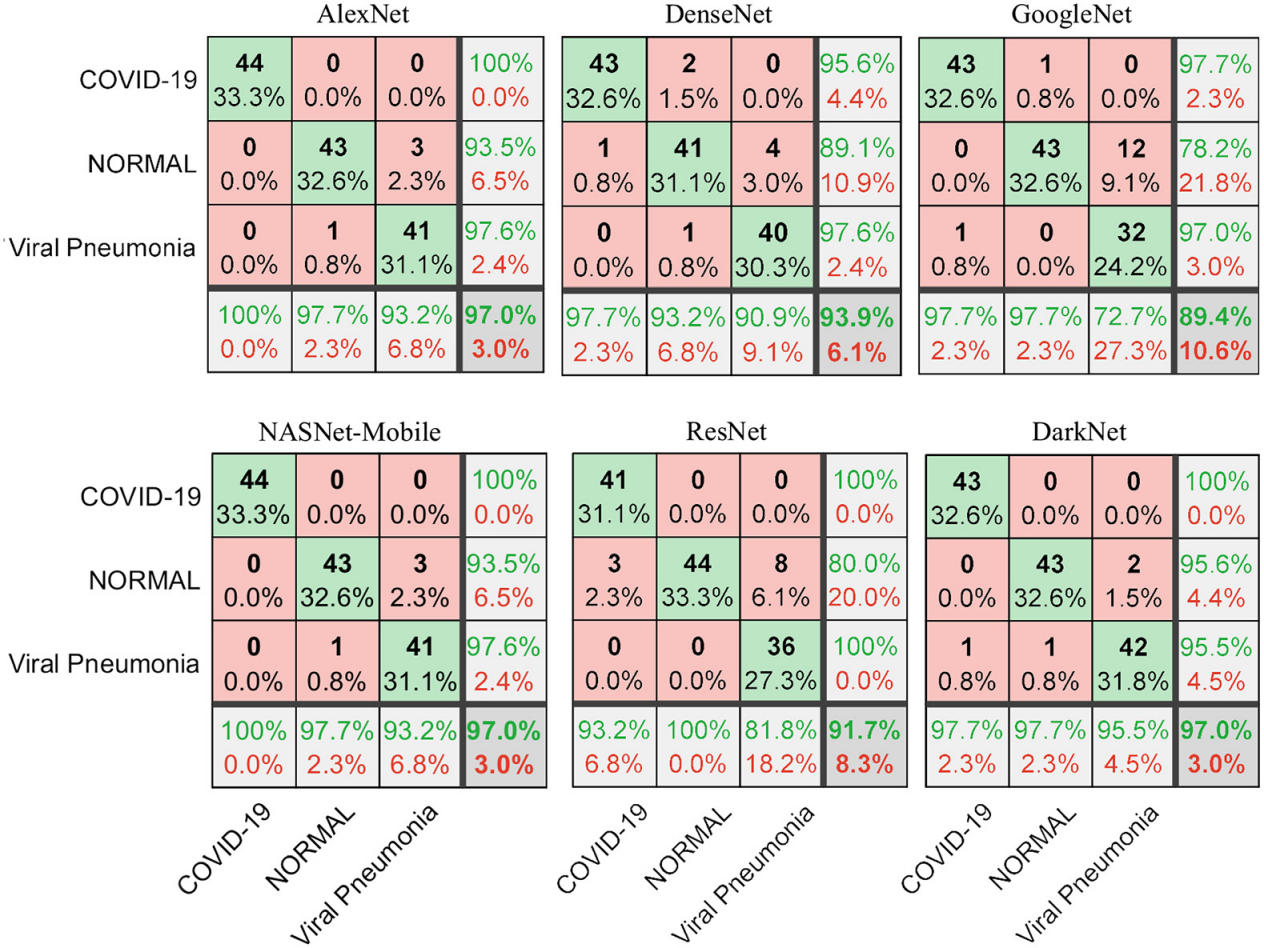
The confusion matrix of testing datasets (20%) shows the performance of correctly classified COVID-19 from normal and pneumonia x-ray images.

Figure 4 shows the confusion matrix of the six DTL models on the task of distinguishing COVID-19 (n=44) from normal (n=44) and pneumonia (n=44) x-ray images in the test set (20%). We note that AlexNet and NASNet-Mobile yield the highest accuracy of 97% for predicting all three classes and of 100% in differentiating COVID-19 samples from normal or pneumonia classes.

**Fig. 5:**
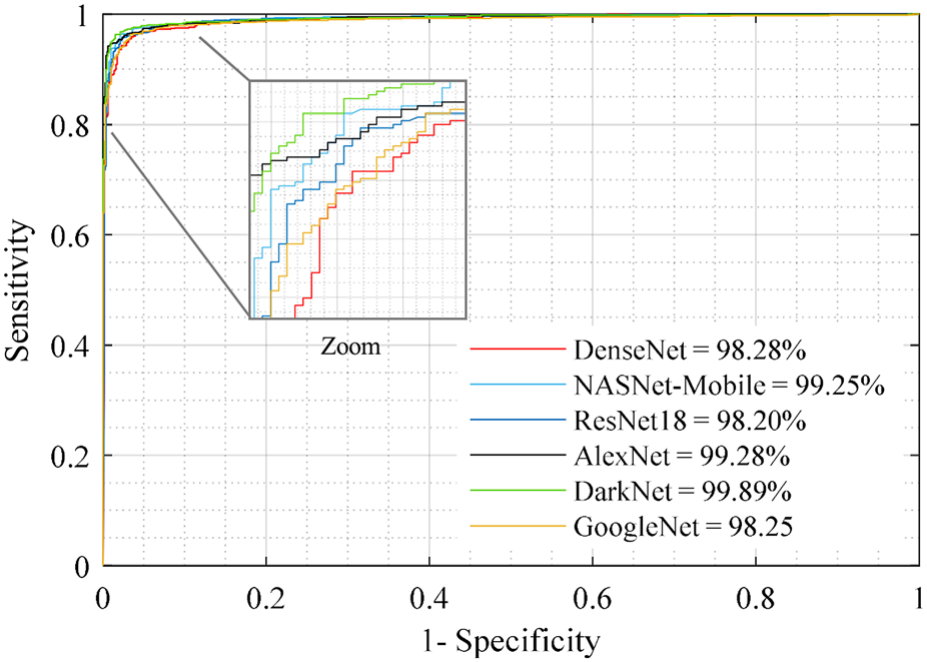
ROC-AUC curve for predicting the COVID-19 CT + x-ray image using DTL models.

To measure the impact of DTL in COVID-19 analysis, we combined CT and x-ray images and grouped these images into two groups: COVID-19 (CT: 276 patients + x-ray: 219 patients) and non-COVID-19 (CT: 397 patients + x-ray: 219 normal subjects + 219 pneumonia patients). We applied five-fold cross-validation (CV) for predicting the COVID-19 images (CT + x-ray). Splitting in each CV fold is based on patients to avoid sharing similar images between training and testing sets. We used 15% of training examples in each fold as validation set to choose hyperparameters. We then measured the average accuracy and AUC values across the five folds (Table 2 and Fig. 5). DTL models show accuracy and AUC value range of 96.66% to 99.09% and 98.12% to 99.89%, respectively. We see that the DarkNet architecture shows the highest accuracy and AUC value of 99.09% and 99.89%, respectively with corrected p<0.05 (Table 3).

**Table 2:** Average of five folds CV for predicting COVID-19 from other viral pneumonia.

CNNs	Accuracy	AUC
AlexNet	97.04	99.28
GoogleNet	96.84	98.25
DenseNet	96.66	98.12
NASNet-Mobile	98.72	99.25
DarkNet	99.09	99.89
ResNet18	96.80	98.20

**Table 3:** Corrected p-value between CNN classifiers for predicting COVID-19 from other viral pneumonia.

CNNs	AlexNet	GoogleNet	DenseNet	NASNet-Mobile	DarkNet	ResNet18
AlexNet	—	—	—	—	—	—
GoogleNet	0.21	—	—	—	—	—
DenseNet	0.24	0.53	—	—	—	—
NASNet-Mobile	0.08	0.04	0.04	—	—	—
DarkNet	0.03	0.02	0.02	0.08	—	—
ResNet18	0.44	0.45	0.43	0.03	0.04	—

Table 4 compares our results with those of previous works. Our approach yields a higher performance compared to existing litterature, with a ∼3% increase in accuracy using x-ray scans.

**Table 4:** Summary of CNN performance metrics (%) for COVID-19 diagnosis using the CT (or/and x-ray) scans.

AI models		Accuracy	AUC	Imaging
Yang et al.^55^		89.00	98.00	CT
Loey et al.^56^		82.91	—	CT
Maghdid et al.^57^		94.10 to 94.00	—	CT + x-ray
Li et al.^14^		—	96.00	CT
Our work (i.e., DarkNet)	Training/validation/test	82.80^a^	90.00	CT
		97.00^b^	—	x-ray
	Five-fold CV	99.09^a^	99.89	CT + x-ray

a 2 classes: COVID-19 versus non-COVID-19.

b 3 classes: COVID-19 versus normal versus pneumonia.

## 4 Discussion

The diagnostic value of chest CT and x-ray is mainly related to the detection of abnormal tissues (lesions) that are not missed by radiography in the early stage. Prediction of these abnormalities will help characterize lesions for further clinical classification and treatment. In this context, deep learning algorithms can be used to improve radiologists’ sensitivity in COVID diagnosis. Specifically, these algorithms have recently demonstrated their potential for screening and detecting COVID-19 in CT and x-ray images.^14^^,^^58^^–^^61^ So far, these studies demonstrate the importance of artificial intelligence in facilitating the prediction of COVID-19 using CT and x-ray images.^60^^,^^62^^–^^68^

We considered DTL models as a non-invasive technique to detect the presence of COVID-19. Our results indicate that these models can differentiate COVID-19 in CT and x-ray test images from non-COVID-19 tissue with the highest accuracy of >82.80 and 97%. Using five-fold CV, DarkNet model demonstrated the highest performance metrics with an accuracy and AUC value of 99.09% and 99.89%, respectively. This finding is consistent with previous studies that considered deep learning to predict, detect, and screen COVID-19 patients.^26^^,^^59^^,^^60^^,^^69^ For example, pretrained CNNs (ResNet50, Inception V3 and Inception-ResNetV2) have shown an accuracy >87% for predicting the COVID-19 using chest x-ray images. Also using deep learning, an AUC of 99.4% was achieved to detect the COVID-19 from non-COVID-19 in Ref. 29. Likewise, a modified pretrained AlexNet model applied on x-rays and CT images obtained an accuracy of 94%.^57^ In Ref. 69, a CNN model with 17 convolutional layers achieved an accuracy of 87% for multiclass classification (COVID-19 versus normal versus other pneumonia). In Ref. 70, a deep model for COVID-19 detection (COVID-Net) gave 92.4% accuracy in classifying normal, non-COVID pneumonia, and COVID-19 classes. Comparing between the CT and x-ray findings, the results in Ref. 22 suggest that chest x-ray could be helpful in monitoring and prognosis but is not recommended for screening.

Comparing with previous studies, our findings show the importance of ROIs in predicting the COVID-19, namely, regions corresponding to consolidation. These are promising results to detect, classify, and predict COVID-19 despite the small number of images used. Furthermore, our results also demonstrate the usefulness of transfer learning algorithms for extracting multiscale texture/patterns in COVID-19 CT images.^71^

So far, AI algorithms applied on COVID-19 chest CT and x-ray scans have shown a potential to improve diagnosis by reducing the subjectivity and variability.^72^ The detection of common findings such as GGO, consolidation, and crazy-paving appearance^73^ can also be impacted by the timing of examining, within or after the patients’ symptoms, and by pre-existing clinical characteristics of the patient. For example, it was found that patients with negative findings in initial chest CT scans would later have rounded peripheral GGO in follow-up scans.^12^ Similar observations were made in Refs. 8 and 74. Moreover, as reported in Refs. 18 and 75, the appearance of GGO and consolidations may vary over time, explaining the discrepancy in sensitivity. Other studies report high sensitivity in diagnosing COVID-19 from CT scans. In addition, some studies have demonstrated the usefulness of CT scans to monitor the abnormality of asymptomatic COVID-19 patients.^76^^,^^77^ For instance, 58 asymptomatic cases with COVID-19 showed abnormal CT findings, predominantly GGO, which were confirmed with nucleic acid testing.^77^^,^^78^ On the other hand, Kim et al.^79^ show that the chest CT screening of patients with suspected disease had a low positive predictive value (range, 1.5% to 30.7%).

This current work has some limitations that could be addressed in future work. We only considered 800 chest CT and 657 chest x-ray images, however, including a larger cohort from different regions of the world could help get a more comprehensive understanding of COVID-19. Moreover, clinical demographics of patients, including age, sex, treatments, and overall survival, were not available for every case and thus not considered in this study.

## 5 Conclusions

We proposed to investigate and develop six models based on DTL that use CT and x-ray images to predict COVID-19. Our results showed that using ROIs of consolidation, GGO and PE in CT images yields the highest accuracy in predicting COVID-19. Furthermore, our findings suggest that DTL models applied on CT and x-ray images could be used as an effective tool for predicting patients who may have contracted the COVID-19. Specifically, DarkNet model is the best DTL model to predict the COVID-19 image. With these automatic models, future studies could reveal additional insights on radiomic markers to assess COVID-19 progression, thereby contributing toward an improved diagnosis and treatment for this disease.
